# Current Perspective in the Discovery of Anti-aging Agents from Natural Products

**DOI:** 10.1007/s13659-017-0135-9

**Published:** 2017-05-31

**Authors:** Ai-Jun Ding, Shan-Qing Zheng, Xiao-Bing Huang, Ti-Kun Xing, Gui-Sheng Wu, Hua-Ying Sun, Shu-Hua Qi, Huai-Rong Luo

**Affiliations:** 1 State Key Laboratory of Phytochemistry and Plant Resources in West China, Kunming Institute of Botany, Chinese Academy of Sciences, Kunming, 650201 Yunnan China; 2 University of Chinese Academy of Sciences, Beijing, 100039 China; 3 Key Laboratory for Aging and Regenerative Medicine, Department of Pharmacology, School of Pharmacy, Southwest Medical University, Luzhou, 646000 Sichuan China; 4 Guangdong Key Laboratory of Marine Material Medical, South China Sea Institute of Oceanology, Chinese Academy of Sciences, Guangzhou, 510301 Guangdong China; 5 Yunnan Key Laboratory of Natural Medicinal Chemistry, Kunming Institute of Botany, Chinese Academy of Sciences, 134 Lanhei Road, Kunming, 650201 Yunnan China

**Keywords:** Aging, Natural products, Anti-aging, Drug screening

## Abstract

Aging is a process characterized by accumulating degenerative damages, resulting in the death of an organism ultimately. The main goal of aging research is to develop therapies that delay age-related diseases in human. Since signaling pathways in aging of *Caenorhabditis elegans* (*C. elegans*), fruit flies and mice are evolutionarily conserved, compounds extending lifespan of them by intervening pathways of aging may be useful in treating age-related diseases in human. Natural products have special resource advantage and with few side effect. Recently, many compounds or extracts from natural products slowing aging and extending lifespan have been reported. Here we summarized these compounds or extracts and their mechanisms in increasing longevity of *C. elegans* or other species, and the prospect in developing anti-aging medicine from natural products.

## Introduction

Since realizing the inevitability of death, the fear of death and pursuit of immortality might have preoccupied with human beings. In the Epic of Gilgamesh, Gilgamesh (the Sumerian king of Uruk) was obsessed in pursuit of immortality herbal. About 200 BC, Qin Shi Huang (the first emperor of a unified China) feared death and desperately sought the fabled elixir of life. A more recent story was the Spanish explorer Ponce de Leon who was looking for the mythical fountain of youth. Unexpectedly, all these human activities of pursuing for immortality were failed. We now know that there is no such elixir of immortality placed in somewhere by god and waited for human to find it.

On the other hand, early medical practice was developed in Babylon, Egypt, Greece, India, and China. Along with the development of biology, chemistry, physics and math, the west medical tradition developed into modern medical science. Great success has been achieved in prevention and treatment of disease. Consequently, the longevity of human has been greatly extended. The aged population is growing rapidly in modern world. Aging is the most risk factor for many age-associated diseases, such as neurodegenerative disease, diabetes, stroke, and cancer. The aged people are often suffering from one or multiple aging associated diseases, which brings enormous social and economic burden. While current medicine is focused on treatment of individual disease, the aging people recovered for one disease would probably suffer from other disease soon later.

Two thousand years ago, a systematic theory and practice to achieve healthy aging with core idea of “preventive treatment of disease” was proposed in Huang Di Nei Jing (one of the most important classical texts of traditional Chinese medicine). Current geroscience research have revealed key molecular processes that underlie biological aging [1], and that delaying aging process could delay the onset and progress of age-associated diseases and the disability of aging people [2]. As the modern version of “the preventative treatment of disease”, anti-aging medicine could be the most effective way to combat the age-associated diseases and the disability of aging people. Currently, many compounds with anti-aging activity have been discovered. A large portion of these compounds are natural products. Therefore, we summarized these natural products or extracts that are reported to have anti-aging effects. We also discussed the prospect and challenges of natural products in development of anti-aging medicine.

## Current Progress in Aging Research

**Fig. 1:**
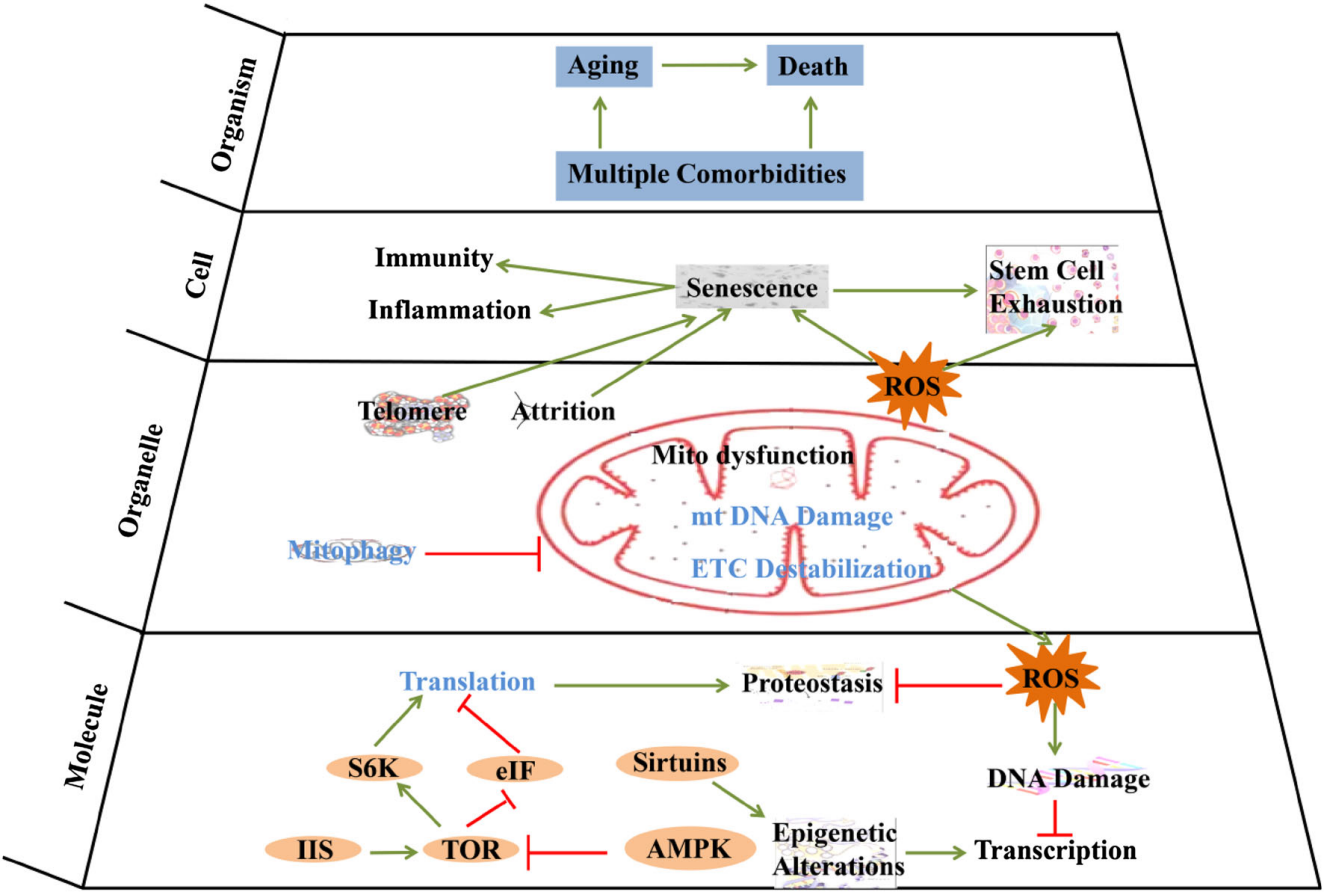
Aging mechanisms in different hierarchies

**Fig. 2:**
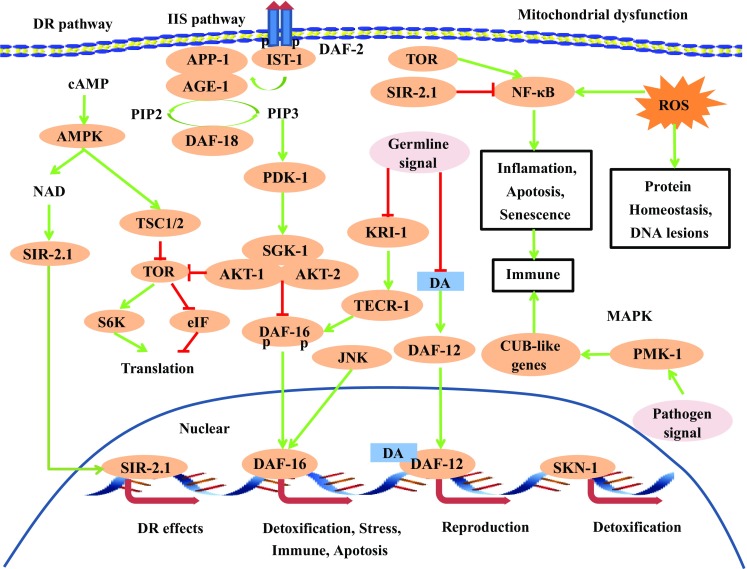
Signaling networks in aging. Dietary restriction (DR), insulin/IGF-1-like signaling (IIS), germline, MAPK and mitochondrial dysfunction pathway networks in aging

Biological process was relying on the delicate interaction of biomolecules. These building blocks of organism were selected during the origin of life, and were imperfect and intrinsic to generation of damage in every biological process, such as in DNA replication, epigenetic modification, transcription and translation, protein post-translational modification, protein fold, and metabolic process. For some of the damages were endangering species survival, their correction mechanisms were evolved by natural selection, such as DNA repair, protein unfolding response, antioxidant mechanism, detoxification, autophagy, and proteasome. The failure of these protection processes would cause the occurrence of aging and pathological phenotypes, while enhancing these protection processes would delay aging and related phenotypes. Here we summarized how the dynamic interactions between various damages and errors occurred in biological process and their evoked response of correction mechanisms contribute to genome stability, proteostasis and metabolic homeostasis, to cellular homeostasis and finally to aging process (Figs. 1, 2). For the detailed mechanisms of aging, we refer to the reviews elsewhere [1, 3–8].

### Genome Stability and Aging

Accumulation of genome damage is one of the major causes of aging [9]. The intrinsic threats to DNA integrity, including DNA replication errors, spontaneous hydrolytic reactions, and reactive oxygen species (ROS), together with exogenous physical (e.g. UV/IR radiation), chemical and biological agents (e.g. virus) cause various genetic lesions, such as point mutations, translocations, chromosomal gains and losses, telomere shortening, and gene disruption. About 70,000 lesions per day were estimated to happen in each normal human cell [10]. Accordingly, a complex repair mechanisms, such as base excision repair (BER), nucleotide excision repair (NER), transcription-coupled repair (TCR), homologous recombination, nonhomologous end-joining (NHEJ), and telomere elongation have been evolved in the organism. The deletion of genes for BER were lethal in mice [11], while mutations affecting NER and TCR were associated with numerous disorders and accelerated aging [12–14]. Mice with defected in NHEJ were subjected to early onset of aging [15]. The discovery of the causality between telomere shortening and cell replication limits, has led to the generation of telomere theory of aging [16]. Patients with inherited telomere syndrome presents greater overall telomere attrition and premature aging [16]. Compounds with improving telomerase activity or suppressing telomere shortening play distinct roles in anti-aging [17].

The epigenetic changes are one of the hallmarks of aging, including alterations in transcription factor binding, histone marks, DNA methylation, and nucleosome positioning [18]. These epigenetic changes can either happen spontaneously or modulated by environmental stimuli, nutrient signaling, and metabolic state, via multiple enzymatic systems including DNA methyltransferases, histone acetylases, deacetylases, methylases, demethylases, and other protein complex. These epigenetic changes can cause aberrant transcription and noncoding RNA expression and impair DNA integrity, affect cellular function and stress resistance, heavily influence the progression of aging. Diet or environment and genetic influencing epigenetic information could alter aging process [19]. Humans and mice with genetic defects in genome maintenance present accelerated aging symptoms, while enhancing DNA maintenance could delay aging [20].

### Proteostasis and Aging

Errors happen on proteins including abnormally synthesized proteins, protein unfolding, abnormal cleavage, undesirable posttranslational modifications, can cause protein self-assembling into toxic oligomeric structures or aggregation into cytosolic inclusions. These damaged proteins can be recognized by chaperones or heat shock proteins and delivered to degradation by the ubiquitin/proteasome system or the lysosomes/autophagy. Increased protein damages would compromise endo-reticulum (ER) homeostasis, lead to increased synthesis of ER chaperones and reduced protein translation to maintain proteostasis, this response is called the unfolding protein response (UPR) [21]. The ability to maintain the protein homeostasis decline with age, many age-related diseases, such as Alzheimer’s disease, Parkinson’s disease, and ALS are associated with intracellular accumulation of abnormal proteins in the form of protein inclusions and aggregates [22]. Chaperone defective could lead to accelerated aging [23], while activation of the master regulator of the heat-shock response, the transcription factor HSF-1, could upregulate heat-shock proteins and increase longevity in *C. elegans* and mice [24, 25].

### Metabolic Homeostasis and Aging

Metabolism provides energy for cell activity, molecules attending signaling transmission, and building block of cell components. Genome instability, proteostasis failure, and environmental influence could lead to abnormal energy supply and metabolite production, such as excessive free oxygen radicals and toxic molecules. Free oxygen radicals including reactive oxygen species (ROS) and diffusible hydrogen peroxide (H_2_O_2_), could lead to accumulated oxidative damages, such as carbonylation, oxidized methionine, glycation, aggregation of proteins and DNA damage, and contribute to aging and age-related diseases [26]. This process was proposed by the famous free radical theory of aging. Many compounds increase longevity or improve age-related diseases via scavenging free radicals, such as resveratrol, astaxanthin and gallic acid [27–29].

JNK, a MAP kinase family member, activated by oxidative stress increases longevity in fruit flies and worms [30, 31]. Reduced function of electron transport chain (ETC) could dramatic extend the lifespan of *C. elegans* and *Drosophila* [32, 33]. Recently research shows that mitophagy modulates bioenergetics and survival in the neurodegenerative disease by reducing redox and damage [34].

The regulation of metabolism is closely coupled with nutrient sensing pathways, including insulin-like growth factor (IGF) signaling (IIS) pathway [35], target of rapamycin (TOR) signaling [36], adenosine monophosphate activated protein kinase (AMPK) pathway [37], and sirtuins [38]. These signaling pathways sense nutrient or metabolites to regulate the level of glucose, amino acid, cAMP and nicotinamide adenine dinucleotide (NAD^+^). These pathways regulate growth, metabolic and aging process. Genetic or pharmacological intervention of their components can extend lifespan and delay age-associated dysregulation [8].

### Cellular Homeostasis and Aging

Failure to maintain genome stability, proteostasis and metabolic homeostasis will lead to imbalance of cellular homeostasis and cellular senescence. Genome instability could lead to abnormality of nuclear structure, while excessive protein aggregation could cause ER malfunction. Genome damage, defective proteins, and excessive production of ROS could impair mitochondria. Mitochondria damage could induce rescue mechanisms: mitochondrial biogenesis, mitochondria specific unfolded protein response and mitophagy (macroautophagy that targets deficient mitochondria for proteolytic degradation) [39]. Recently research shows that mitophagy modulates bioenergetics and survival in the neurodegenerative disease by reducing redox and damage [34]. The increased damage and reduced repair response are important to aging process.

Senescent cells secret signaling molecules enriched in proinflammatory cytokines and matrix metalloproteinases, which could attract mast cells to clear the senescent cells through macrophage. But deficient clearance of senescent cells will induce inflammation, impair adjacent cells and tissue function, and lead to stem cell exhaustion, and finally contribute to aging [40]. Either genetic or pharmacological elimination of senescent cells could delay age-related pathologies [41, 42].

## Natural Products with Anti-aging Activity

To date, there are about 5, 400 scientific research/review articles published under the terms of “anti-aging” and “anti-ageing” terms (obtained from Web of Science, May 2017; keywords restricted to the topics: anti-aging and anti-ageing, at the search domain of Science & Technology). These reports revealed more than 300 compounds with anti-aging activity. Here we summarized the compounds or natural product extracts with explicit anti-aging activity, including 185 compounds from natural products (Table 1), 55 complex or extracts from natural products (Table 2), 62 from clinical drugs (of which more than 50% are also from natural products or natural products analogues, Table 3), 35 from synthesized chemicals (Table 4). Some of them received popular interest and under vigorous investigation, present anti-aging activities in multiple aging models, such as resveratrol [28, 43–53], α-lipoic acid [54–56], astaxanthin [29, 57–59], catechin [60–62], curcumin [63–65], fucoxanthin [66, 67], spermidine [68, 69], metformin [70–72], caffeine [73–75], and rapamycin [76–84], all show anti-aging activity in both *D. melanogaster* and *C. elegans*, as well as in other aging models (Table 1). There are 39 compounds present anti-aging activity in two aging models, 32 of them with anti-aging activity in *C. elegans*. 19 of the 39 compounds are antioxidant (including acacetin, antcin M, agmatine, baicalein, caffeic acid, carnosine, chlorogenic acid, coenzyme Q10, dimethyl sulfide, gallic acid, gluconate, glycerol, hesperidin, icariin, lactate, oleanolic acid, minocycline, vitamin E, and vitexin). Compound betaine, catalpol, (−)-epicatechin, huperzine A and polydatin regulate inflammation. 11 compounds act through energy sensing pathway, including acetic acid, α-ketoglutarate, D-glucosamine, epigallocatechin gallate, nordihydroguaiaretic acid, oligonol, polydatin, rosmarinic acid, sesamin, aspirin, and tetrahydrocurcumin. There are 14, 9, and 109 natural products with anti-aging activity reported only in mice or rat, fruit fly, and *C. elegans*, respectively, while 14 compounds present anti-aging activities in other aging models, such as mammalian cells and *S. cerevisiae*. Among the 109 compounds with anti-aging activity in *C. elegans*, 18 with antioxidative activity, five regulating IIS pathway, four regulating AMPK, four regulating mTOR signaling, 10 regulating SIR-2.1, six regulating SKN-1/Nrf2 pathway, seven regulating JNK-1, 16 with unknown mechanisms, and about half of 109 compounds revealed to regulate multiple signaling pathways.

**Table 1:** Compounds from natural products with anti-aging activities

CAS	Chemicals		Structure	Source	Anti-aging activity and proposed anti-aging mechanism
With anti-aging activities in a variety of aging models					
501-36-0	Resveratrol			*Polygonum cuspidatum Sieb.et Zucc.*	In mice: 4.7% increase in mean lifespan; increasing insulin sensitivity, reducing insulin-like growth factor-1 (IGF-I) levels, increasing AMP-activated protein kinase (AMPK) and peroxisome proliferator-activated receptor-gamma coactivator 1alpha (PGC-1alpha) activity, increasing mitochondrial number, and improving motor function [44, 46–51] In *D. melanogaster*: extends mean lifespan of females fed the low sugar–high protein diet by ∼15.0%, fed the high-fat diet by ∼10.0%; modulating genetic pathways that can reduce cellular damage [45] In *C. elegans*: 18.0% increase in mean lifespan; regulating AMPK, SIR-2.1, autophagy, and proteasomal degradation [28, 52, 53] In cell: increasing NAD(+) and the activity of AMPK and Sirt1, inhibiting PDE4, JAK2/STAT3 [89–92] In *S. cerevisiae*: 70.0% increase in mean lifespan; regulating Sir2 and SNF1 [93, 94] In *Nothobranchius guentheri*: antioxidant [95]
62-46-4	α-Lipoic acid			Cell metabolite	In SAMP8 mice: improving memory and oxidative stress in extremely old SAMP8 mice, but decreasing lifespan [56] In *D. melanogaste*: 12.0% increase in mean lifespan and antioxidant [54] In *C. elegans*: 24.0% increase in mean lifespan and antioxidant, enhancing chemotaxis index [55]
472-61-7	Astaxanthin			Carotenoid	In d-galactose-induced brain aging in rats: antioxidant, upregulating BDNF expression [58, 59] In *D. melanogaster*: antioxidant [57] In *C. elegans*: 29.0% increase in mean lifespan and regulating DAF-16 [29]
154-23-4	Catechin			Green tea, cocoa, grapes, and apples	In senescence-accelerated (SAMP10) mice: preventing memory regression and DNA oxidative damage [62] In *D. melanogaster*: 16.0% increase in mean lifespan and antioxidant [61, 96] In *C. elegans*: 13.0% increase in mean lifespan and antioxidant, regulating DAF-2, AKT-2, MEV-1, and NHR-8; decreasing insulin-like growth factor-1 [60]
458-37-7	Curcumin			*Curcuma longa* L.	In C57BL6/N mice: antioxidant, increasing collagen and AGEs [64] In *D. melanogaster*: 25.8% increase in mean lifespan and antioxidant [65] In *C. elegans*: 25.0% increase in mean lifespan and antioxidant [63]
3351-86-8	Fucoxanthin			Natural substances in human diet	In hairless mice: lessening UVB-induced epidermal hypertrophy, VEGF, and MMP-13 expression [67] In *D. melanogaster*: 33.0% increase in mean lifespan and antioxidant [66] In *C. elegans*: 14.0% increase in mean lifespan and antioxidant [66]
124-20-9	Spermidine			Natural polyamine	In *D. melanogaster*: 30.0% increase in mean lifespan and autophagy [68] In *C. elegans*: 15.0% increase in mean lifespan and autophagy [68] In *S. cerevisiae*: autophagy [68, 69]
With anti-aging activities in two aging models					
480-44-4	Acacetin			Naturally occurring flavonoid	In *D. melanogaster:* decreasing APP protein expression, BACE-1 activity, and Aβ production [97] In *C. elegans*: 27.3% increase in mean lifespan and upregulating SOD-3 and GST-4 [98]
64-19-7	Acetic acid			Vinegars	In *C. elegans*: 23.0% increase in mean lifespan and regulating insulin/IGF-1 pathway [99] In *S. cerevisiae*: stimulating growth signaling pathways, increasing oxidative stress and replication stress [100]
1005344-44-4	Antcin M			*Antrodia cinnamomea*	In cell: antioxidant, regulating Nrf2 and SIRT-1 [101] In *C. elegans* from oxidative stress: ~10.0% increase in mean lifespan and antioxidant [101]
306-60-5	Agmatine			Generated by arginine decarboxylase	In male sprague–dawley rats: suppressing age-related elevation in nitric oxide synthase activity in the dentate gyrus of the hippocampus and prefrontal cortex [102] In *C. elegans*: 16.0% increase in mean lifespan and needs further research [103]
328-50-7	α-Ketoglutarate			Tricarboxylic acid cycle intermediate	In mice: decreasing TBARS level and the activity of superoxide dismutase, increasing glutathione peroxidase activity [104] In *C. elegans*: 50.0% increase in mean lifespan and inhibiting ATP synthase and TOR signaling [105]
491-67-8	Baicalein			*Scutellaria baicalensis* Lamiaceae	In *C. elegans*: 24.0% increase in mean lifespan and antioxidant, regulating SKN-1 [106] In PC12 cell: suppressing mitochondria dysfunction and apoptosis [107]
107-43-7	Betaine			Nitrogen containing metabolite	In aged rats: upregulating IKK/MAPKs, attenuating NF-κB activation [108, 109] In *C. elegans*: 9.0% increase in mean lifespan and needs further research [103]
331-39-5	Caffeic acid			Tomatoes, carrots, strawberries, blueberries and wheat	In Sprague–Dawley rats and intra-cerebroventricular streptozotocin induced experimental dementia in rats: antioxidant, restoring cholinergic functions [110, 111] In *C. elegans*: 11.0% increase in mean lifespan and regulating OSR-1, SEK-1, SIR-2.1, UNC-43, and DAF-16 [112]
305-84-0	Carnosine			Endogenous dipeptide	In aged rats: preventing oxidative stress and apoptosis [113, 114] In *D. melanogaster:* 26.0% increase in mean lifespan and antioxidant [115, 116]
2415-24-9	Catalpol			*Rehmannia glutinosa*	In senescent mice induced by d-galactose: improving cholinergic function, reducing inflammatory cytokines; In rats: rebalancing E2 and P4 levels in aged rats [117, 118] In *C. elegans*: 28.5% increase in mean lifespan and antioxidant, regulating SKN-1/Nrf and DAF-16 [119]
327-97-9	Chlorogenic acid			Coffee and tea	In d-galactose-induced mice: antioxidant, reducing tumour necrosis factor-α (TNF-α) and interleukin-6 (IL-6) protein levels [120] In *C. elegans*: 20.1% increase in mean lifespan and antioxidant, regulating IIS pathway [121]
303-98-0	Coenzyme Q10			Mitochondrial respiratory chain component	In mice: ameliorating age-related impairment, reducing protein oxidation [122] In *C. elegans*: 18.0% increase in mean lifespan and scavenging reactive oxygen species [123]
3416-24-8	d-Glucosamine			Hexosamine pathway	In mice: 6.0% increase in mean lifespan; enhancing expression of several murine amino-acid transporters, increasing amino-acid catabolism [124] In *C. elegans*: 11.0% increase in mean lifespan and mimicking a low-carbohydrate diet by regulating AMPK and SKN-1 [124]
75-18-3	Dimethyl sulfide			Metabolite of marine algae or fermentative bacteria	In *D. melanogaster*: 24.2% increase in mean lifespan and antioxidant [17] In *C. elegans*: 24.3% increase in mean lifespan and antioxidant [17]
490-46-0	(−)-Epicatechin			Cocoa	In obese diabetic mice: antioxidant, improving skeletal muscle stress output, reducing systematic inflammation and serum LDL cholesterol [61] In *D. melanogaster*: ~8.0% increase in mean lifespan and needs further research [61]
989-51-5	Epigallo-catechin gallate			Tea polyphenols	In d-galactose-induced mice: increasing oxidative stress and the expression of EGFR proteins [125] In *C. elegans*: 13.0% increase in mean lifespan and antioxidant, regulating IIS pathway [126]
149-91-7	Gallic acid			Beverages (red wines and green teas), plant leaves (*beriberry*)	In senescence accelerated mice: antioxidant [127] In *C. elegans*: 25.0% increase in mean lifespan and antioxidant [27]
527-07-1	Gluconate			Sugars metabolite	In *D. melanogaster*: 22.0% increase in mean lifespan and antioxidant [128] In lacking nitrogen *on C. elegans*: 16.0% increase in mean lifespan and antioxidant [103]
56-81-5	Glycerol			Sugars metabolite	In lacking nitrogen *on C. elegans*: 21.0% increase in mean lifespan and needs further research [103] In rotifer: 50.0% increase in mean lifespan; increasing resistance to starvation, heat, oxidation, and osmotic stress, but not UV stress [129]
520-26-3	Hesperidin			*Citrus* genus	In Murine model of sepsis: antioxidant [130] In *S. cerevisiae*: 37.0% increase in mean lifespan and antioxidant, regulating Sir2, UTH1 [131]
489-32-7	Icariin			*Herba epimedii*	In mice: inducting antioxidant protein superoxide dismutase (SOD) activity, decreasing oxidative marker malondialdehyde (MDA) [132] In *C. elegans*: 20.7% increase in mean lifespan and regulating IIS pathway [133]
74-79-3	Arginine			Amino acid	In *C. elegans*: 27.0% increase in oxidative stress; 370% in heat stress and antioxidant, regulating insulin/IGF signaling pathway [103, 134] In *Megalobrama amblycephala*: antioxidant [134]
50-21-5	Lactate			Metabolite	In *D. melanogaster*: 15.0% increase in mean lifespan and antioxidant [128] In *C. elegans*: 6.0% increase in mean lifespan and antioxidant [103]
500-38-9	Nordi-hydroguaiaretic acid			Creosote plant (*Larrea tridentata: Zygophyllaceae*)	In mice:12.0% increase in mean lifespan; decreasing the absorption or increasing the utilization of calories [135–138] In Mosquito: 64.0% increase in mean lifespan and needs further research [137]
508-02-1	Oleanolic acid			*Olea europaea, Viscum album* L., and *Aralia chinensis* L.	In d-galactose-induced mice: anti-oxidative, anti-glycative, and anti-apoptotic [139] In *C. elegans*: 16.6% increase in mean lifespan and antioxidant, regulating DAF-16 [140]
851983-55-6	Oligonol			Grape seed or lychee fruit	In mice: Regulating AMPK, SIRT1, autophagy, and increasing cell proliferation [141, 142] In *C. elegans*: regulating AMPK and autophagy [141]
27208-80-6	Polydatin			Grape juice	In mice: anti-oxidative, anti-inflammatory, and anti-apoptotic [143] In *C. elegans*: 30.0% increase in mean lifespan and regulating DAF-2, SIR-2.1, SKN-1, SOD-3, and DAF-16 [144]
537-15-5	Rosmarinic acid			Subfamily *Nepetoideae* of the *Lamiaceae*	In aging mice: antioxidant [145] In *C. elegans*: 10.0% increase in mean lifespan and regulating SIR-2.1, OSR-1, SEK-1, UNC-43, and DAF-16 [112]
607-80-7	Sesamin			Sesame seeds	In *D. melanogaster:* 12.0% increase in mean lifespan and antioxidant [146] In *C. elegans*:14.0% increase in mean lifespan and regulating DAF-2, SKN-1, PMK-1, and DAF-16 [147]
36062-04-1	Tetra-hydrocurcumin			Biotransformed metabolite of curcumin contained in turmeric of Indian curry	In mice: 12.0% increase in mean lifespan; attenuating oxidative stress, hypertension, vascular dysfunction, and baroreflex dysfunction [148–151] In *D. melanogaster*: ~28.0% increase in mean lifespan and regulating Sir2 and FoxO [152]
1143-70-0	Urolithin A			*Pomegranate* fruit, nuts and berries	In mouse models of age-related decline of muscle function: improving exercise capacity [153] In *C. elegans*: 45.4% increase in mean lifespan and regulating mitochondrial function, mitophagy [153]
3681-93-4	Vitexin			*Vigna angularis*	In d-galactose-aged mice: antioxidant [154] In *C. elegans*: 17.0% increase in mean lifespan and antioxidant [155]
With anti-aging activities in rats or mice					
118-00-3	Guanosine			Endogenous nucleoside	In Wistar rats: antioxidant [156]
70579-26-9	Porphyra-334			Red alga *Porphyra rosengurttii*	In mice skin: antioxidant, Hsp70 [157]
73112-73-9	Shinorine			Red alga *Porphyra rosengurttii*	In mice skin: antioxidant, Hsp70 [157]
70363-87-0	Sargaquinoic acid			*Sargassum sagamianum*	In mice skin: inducing apoptosis [158]
70363-89-2	Sargachromenol			*Sargassum sagamianum*	In mice skin: inducing apoptosis [158]
1094-61-7	β-Nicotinamide mononucleotide			Turnover of the oxidized form of nicotinamide adenine dinucleotide (NAD^+^)	In rats: increasing NAD^+^ level [159]
1339070-29-9	TA-65			Root of *Astragalus membranaceus*	In mice: activating telomerase [160]
34157-83-0	Celastrol			Traditional Chinese medicinal herbs of the *Celastraceae* family	In transgenic mouse model of amyotrophic lateral sclerosis: 13.0% increase in mean lifespan and regulating HSP70, blocking neuronal cell death [161]
57-00-1	Creatine			Natural ergogenic compound	In mice: 9.0% increase in mean lifespan and upregulating genes implicated in neuronal growth, neuroprotection, and learning [162]
42553-65-1	Crocin			Kashmiri saffron (*Crocus sativus*)	In mice: 44.0% increase in mean lifespan and impacting on hematological parameters [163]
61276-17-3	Acteoside			Roots of *Incarvillea younghusbandii Sprague*	In senescent mouse model induced by a combination of d-gal and AlCl3: decreasing nitric oxide, the activity of nitric oxide synthase and the expression of *caspase*-*3* [164]
11096-26-7	Erythropoietin	Glycoprotein		Glycoprotein hormone	In rats: antioxidant, regulating ERK/Nrf2-ARE [165]
62499-27-8	Gastrodin			A number of plants and herbs	In vascular dementia rats induced by chronic ischemia: antioxidant, regulating ADH7, GPX2, GPX3 and NFE2L2 [166]
22427-39-0	Ginsenoside Rg1			*Panax ginseng*	In d-galactose-induced mice: antioxidant, regulating the level of proinflammatory cytokines and telomerase system, activating the Wnt/β-catenin signaling [167, 168]
With anti-aging activities in *Drosophila melanogaster*					
87-89-8	Chiro-inositol			Inositol family	16.7% increase in mean lifespan and antioxidant, regulating dFOXO [169]
526-95-4	Gluconic acid			Glucose catabolism	22.0% increase in mean lifespan and antioxidant [128]
	Glycoside acteoside			Roots of *Incarvillea younghusbandii Sprague*	15.0% increase in mean lifespan and antioxidant [170]
127-40-2	Lutein			Major carotenoids in most fruits and vegetables	11.0% increase in mean lifespan and antioxidant [171]
1004313-10-3	*S*,*S*-Trolox-carnosine			Trolox acylated derivatives	36.0% increase in mean lifespan and antioxidant [116]
4670-05-7	Theaflavins			Black tea	10.0% increase in mean lifespan and antioxidant [172]
353-09-3	β-Guani-dinopropionic acid			Metabolites	13.0% in female, 90% in male increase in mean lifespan and regulating AMPK-Atg1-autophagy signaling [173]
19545-26-7	Wortmannin			*Penicillium funiculosum*	5.0% increase in mean lifespan and inhibiting PI3K [174]
139-85-5	3,4-Dihydro-xybenzaldehyde			*Sasa senanensis* leaves	23.0% increase in mean lifespan and inhibiting the 2-oxoglutarate binding sites of prolyl 4-hydroxylase [175]
With anti-aging activities in *Caenorhabditis elegans*					
57-91-0	β-Estradiol			Hormone	7.0% increase in mean lifespan and antioxidant [176]
1406-65-1	Chlorophyll			Green vegetables	25.0% increase in mean lifespan and antioxidant [177]
730-08-5	Dipeptide Tyr-Ala			Hydrolyzed maize protein	12.4% increase in mean lifespan and antioxidant [178]
934822-64-7	Ferulsinaic acid			Sesquiterpene coumarins from the genus Ferula	20.0% increase in mean lifespan and antioxidant [179]
446-72-0	Genistein			*Vigna angularis*	27.9% increase in mean lifespan and antioxidant [180, 181]
	Quercetin 3-*O*-β-d-glucopyranoside-(4 → 1)-β-d-glucopyranoside			Onion	12.4% increase in mean lifespan and antioxidant [182]
69-72-7	Salicylic acid			Plant hormone	14.0% increase in mean lifespan and antioxidant [183]
72514-90-0	Specioside			*Stereospermum suaveolens*	15.5% increase in mean lifespan and antioxidant [184]
480-18-2	Taxifolin			Citrus fruits and onion	51.0% increase in mean lifespan and antioxidant [185]
3081-61-6	Theanine			*Camellia sinensis*	~5.0% increase in mean lifespan and antioxidant [186]
6829-55-6	Tocotrienols			Vitamin E members	~20.0% increase in mean lifespan and antioxidant [187]
53188-07-1	Trolox			Vitamin E analog	31.0% increase in mean lifespan and antioxidant [185]
528-48-3	Fisetin			Apples, onions and grapes and many more herbal edibles	6.0% increase in mean lifespan of thermal stress and antioxidant, regulating DAF-16 [188]
215112-16-6	4-Hydroxy-*E*-globularinin			*Premna integrifolia*	18.8% increase in mean lifespan and antioxidant, regulating DAF-16 [189]
521-48-2	Iso-xanthohumol			*Humulus lupulus* L.	10.2% increase in mean lifespan and antioxidant, regulating DAF-16 [190]
520-18-3	Kaempferol			Apples, onions and grapes and many more herbal edibles	10.0% increase in mean lifespan and antioxidant,regulating DAF-16 [188]
117-39-5	Quercetin			Onions, apples, and broccoli as well as in red wine, tea, and extracts of *Ginkgo biloba*	15.0% increase in mean lifespan and antioxidant, regulating DAF-16 [191]
50932-19-9	Verminoside			*Stereospermum suaveolens*	20.8% increase in mean lifespan and antioxidant, regulating DAF-16 [192]
113558-15-9	Icariside II			Icariin active metabolite	20.0% increase in mean lifespan and regulating IIS signaling [133]
99-20-7	Trehalose			Disaccharide of glucose	32.0% increase in mean lifespan and regulating IIS signaling [193]
32911-62-9	Withanolide A			*Ayurvedic*	29.7% increase in mean lifespan and regulating IIS pathway and neural activity [194]
501-94-0	Tyrosol			Extra virgin olive oil	10.8% increase in mean lifespan and regulating IIS pathway and heat shock response [195–197]
4339-71-3	Piceatannol			Grapes and white tea	~18.3% increase in mean lifespan and regulating IIS pathway and SIR-2.1 [198]
52-89-1	Cysteine			Amino acids	16.0% increase in mean lifespan and regulating AMPK and DAF-16 [103]
6537-80-0	Chicoric acid			Caffeoyl derivative	21.0% increase in mean lifespan and regulating AMPK [199]
328-42-7	Oxaloacetate			Citric acid cycle metabolite	25.0% increase in mean lifespan and regulating AMPK [200]
29700-22-9	Oxy-resveratrol			Isomer of hydroxylated resveratrol	31.1% increase in mean lifespan and regulating calorie restriction, AMPK, and SIR-2.1 [201]
	β-Dihydro-agarofuran-type sesquiterpenes			Seeds of *Celastrus monospermus*	38.0% increase in mean lifespan and rapamycin mimetics [202]
13095-47-1	(*R*)-2-Hydro-xyglutarate			Oncometabolite	43.0% increase in mean lifespan and inhibiting ATP synthase and mTOR signaling [203]
13095-48-2	(*S*)-2-Hydroxyglutarate			Oncometabolite	32.0% increase in mean lifespan and inhibiting ATP synthase and mTOR signaling [203]
765-01-5	10-Hydroxy-2-decenoic acid			Major lipid component of Royal Jelly	10.0% increase in mean lifespan and regulating dietary restriction and mTOR signaling [204]
	Ascr#2			Pheromone	14.0% increase in mean lifespan and regulating SIR-2.1 [205]
	Ascr#3			Pheromone	14.0% increase in mean lifespan and regulating SIR-2.1 [205]
1740-19-8	Dehydro-abietic acid			*P. densiflora, P. sylvestris, Abies grandis*	15.5% increase in mean lifespan and regulating SIR-2.1 [206]
7783-06-4	Hydrogen sulfide			Naturally produced in animal cells	74.0% increase in mean lifespan and antioxidant, regulating SIR-2.1 [207, 208]
932-30-9	Salicylamine			Phenolic amines	56.0% increase in mean lifespan and regulating SIR-2.1 and ETS-7 [209]
481-39-0	Juglone			Roots, leaves, woods and fruits of *Juglandaceae* walnut trees	29.0% increase in mean lifespan and regulating SIR-2.1 and DAF-16 [210]
	Deutero-haemin-AlaHisThrValGluLys	Peptides		Porphyrin-peptide	19.1% increase in mean lifespan and regulating SIR-2.1and DAF-16 [211]
53-84-9	Nicotinamide adenine dinucleotide			Tricarboxylic acid cycle intermediate	15.0% increase in mean lifespan and regulating SIR-2.1 and DAF-16 [212]
149-61-1	Malate			Tricarboylic acid (TCA) cycle metabolite	14.0% increase in mean lifespan and regulating dietary restriction, SIR-2.1, and DAF-16 [213]
70-47-3	Asparagine			Amino acid	5.0% increase in mean lifespan and regulating SKN-1 [103]
2050-87-5	Diallyl trisulfide			*Garlic*	12.6% increase in mean lifespan and regulating SKN-1 [214]
481-42-5	Plumbagin			*Plumbago zeylanica* L.	15.0% increase in mean lifespan and regulating SKN-1 [215]
77-59-8	Tomatidine			Unripe tomato fruits, leaves and stems	7.0% increase in mean lifespan and regulating SKN-1/Nrf2 pathway, mitophagy [216]
21593-77-1	*S*-Allylcysteine			*Allium sativum* L.	17.0% increase in mean lifespan and antioxidant, regulating SKN-1 [217]
2281-22-3	*S*-Allylmercapto-cysteine			*Allium sativum* L.	20.9% increase in mean lifespan and antioxidant, regulating SKN-1 [217]
61-90-5	Leucine			Amino acids	16.0% increase in mean lifespan and regulating SKN-1and DAF-16 [103]
62333-08-8	Isolappaol A			*A. lappa* seeds	11.0% increase in mean lifespan and regulating JNK-1 and DAF-16 [218]
64855-00-1	Lappaol C			*A. lappa* seeds	12.0% increase in mean lifespan and regulating JNK-1 and DAF-16 [218]
69394-17-8	Lappaol F			*A. lappa* seeds	13.0% increase in mean lifespan and regulating JNK-1 and DAF-16 [218]
580-72-3	Matairesinol			*Arctium lappa*	25.0% increase in mean lifespan and regulating JNK-1 and DAF-16 [218]
7770-78-7	Arctigenin			*Arctium lappa*	14.0% increase in mean lifespan and antioxidant, regulating JNK-1 and DAF-16 [218]
20362-31-6	Arctiin			*Arctium lappa*	15.0% increase in mean lifespan and antioxidant, regulating JNK-1 and DAF-16 [218]
484-68-4	Pinitol			Fine wood, alfalfa, and legumes	13.0% increase in mean lifespan and regulating JNK, S6K, and DAF-16 [169]
56-41-7	Alanine			Amino acid	11.0% increase in mean lifespan and regulating AAK-2, SKN-1, and DAF-16 [103]
56-87-1	Lysine			Amino acids	8.0% increase in mean lifespan and regulating AAK-2, SKN-1, and DAF-16 [103]
338-69-2	d-Alanine			Amino acids	16.0% increase in mean lifespan and regulating AAK-2, SIR-2.1, and DAF-16 [103]
56-85-9	Glutamine			Amino acids	16.0% increase in mean lifespan and regulating EAT-2, AAK-2, and SKN-1 [103]
87-44-5	β-Caryophyllene			Edible plants	22.0% increase in mean lifespan and regulating SIR-2.1, SKN-1 and DAF-16 [219]
60-18-4	Tyrosine			Amino acids	10.0% increase in mean lifespan and regulating SIR-2.1, SKN-1, and DAF-16 [103]
107-95-9	β-Alanine			Amino acid	13.0% increase in mean lifespan and regulating AAK-2, SIR-2.1, SKN-1, and DAF-16 [103]
	Acacetin 7-*O*-α-l-rhamnopyranosyl (1–2) β-d-xylopyranoside			*Premna integrifolia*	39.0% increase in mean lifespan and regulating EAT-2, SIR-2.1, SKN-1, HSF-1, MEV-1, and DAF-16 [220]
15502-74-6	Arsenite			Natural and anthropogenic sources	(10 μM) 10.0% increase in mean lifespan, (>100 μM) 12.0% decrease and antioxidant, regulating SKN-1, MTL-2, TIN-9, and DAF-16 [221, 222]
625-72-9	d-β-Hydroxybutyrate			Ketone body	26.0% increase in mean lifespan and regulating AAK-2, SIR-2.1, SKN-1, and DAF-16; inhibiting histone deacetylase [223]
71-00-1	Histidine			Amino acids	12.0% increase in mean lifespan and regulating EAT-2, AAK-2, SIR-2.1, SKN-1, BEC-1, HIF-1, GAS-1, IFE-2, GCN-2, and DAF-16 [103]
37159-97-0	Proline			Amino acids	19.0% increase in mean lifespan and regulating EAT-2, AAK-2, SIR-2.1, SKN-1, BEC-1, and DAF-16 [103]
56-45-1	Serine			Amino acids	22.0% increase in mean lifespan and regulating EAT-2, AAK-2, SIR-2.1, SKN-1, HIF-1, BEC-1, and DAF-16 [103]
73-22-3	Tryptophan			Amino acids	14.0% increase in mean lifespan and regulating EAT-2, AAK-2, SIR-2.1, SKN-1, BEC-1, GCN-2, and DAF-16 [103]
1405-87-4	Bacitracin			*Bacillus subtilisvar* Tracy	59.0% increase in mean lifespan and regulating CBP-1, improving proteotoxcity [224]
142-42-7	Fumarate			Tricarboxylic acid (TCA) cycle metabolite	16.0% increase in mean lifespan and increasing the amount of oxidized NAD and FAD cofactors [213]
63-68-3	Methionine			Amino acids	14.0% increase in mean lifespan and regulating mitochondrial unfolded protein response [103]
62-75-9	*N*-Nitrosodimethylamine			Ubiquitously distributed organic xenobiotic compounds	6.0% increase in mean lifespan and reducing transcription of many stress response genes [225]
25166-14-7	2,3-Dehydrosily-bin A/B			Potential active components of silymarin	16.1% increase in mean lifespan and antioxidant, regulating FGT-1, improving proteotoxic stress [226]
476-66-4	Ellagic acid			Strawberry and raspberry	~10.0% increase in mean lifespan and antioxidant, CR mimetics, antimicrobial [27]
1259-86-5	Glau-carubinone			Different species of the tropical plant family *Simaroubaceae*	~80.0% increase in mean lifespan and promoting mitochondrial metabolism, reducing body fat [227]
529-44-2	Myricetin			Tea, different vegetables, onions, berries, grapes and medical plants	34.3% increase in mean lifespan and regulating DAF-16; enhanced quality of life during aging [63, 228]
106758-54-7	Otophylloside B			*Cynanchum otophyllum*	11.3% increase in mean lifespan and regulating DAF-2, SIR-2.1, CLK-1, and DAF-16 [229]
14937-32-7	Pentagalloyl glucose			*Eucalyptus* leaves	18.0% increase in mean lifespan and regulating dietary restriction, IIS pathway, SIR-2.1 and mitochondrial electron transport chain [230]
7512-17-6	*N*-Acetyl-glucosamine			Hexosamine Pathway Metabolite	50.0% increase in mean lifespan and enhancing autophagy, ER-associated protein degradation, and proteasomal activity [231]
	Quercetin 3′-*O*-β-d-glucopyranoside			Onion	20.9% increase in mean lifespan and regulating DAF-2, OLD-1, OSR-1,and AEK-1 [182]
50-70-4	Sorbitol			*S. cerevisiae*	35.0% increase in mean lifespan and regulating DR and osmotic response [232]
1401-55-4	Tannic acid			Grapes and green tea	19.0% increase in mean lifespan and regulating TGF-β, p38 MAPK pathways, and DAF-12 [233, 234]
77-92-9	Citrate			Tricarboxylic acid cycle intermediate	13.0% increase in mean lifespan and inducing ER stress response [103]
107-35-7	Taurine			Nitrogen containing metabolites	11.0% increase in mean lifespan and inducing ER stress response [103]
38748-32-2	Triptolide			Tripterygium wilfordii	20.1% increase in mean lifespan and antioxidant, regulating HSP16.2 and SOD-3 [235]
67-97-0	Vitamin D3			Vitamins	39.0% increase in mean lifespan and regulating SKN-1, IRE-1, XBP-1, DAF-12, and proteostasis [236]
57-88-5	Cholesterol			Cyclo-pentanoper-hydro-phenanthrene ring	Regulating cholesterol-binding protein NSBP-1and DAF-16 [237]
949004-12-0	Dafachronic acid			Bile acid-like steroid	17.0% increase in mean lifespan and “antiaging” in the germ-line longevity pathway [238]
145-13-1	Pregnenolone			Hormonal steroids	20.0% increase in mean lifespan and relating to germline-defective regulated longevity [239]
1315285-41-6	Royalactin	Glycoprotein		Royal jelly	34.0% increase in mean lifespan and regulating EGF signaling [240]
104594-70-9	Caffeic acid phenethyl ester			Propolis	9.0% increase in mean lifespan and regulating DAF-16 [241]
64-17-5	Ethanol			Metabolites	Serving as a carbon and energy source and/or by inducing a stress response [242]
	*N*-γ-(l-Glutamyl)-l-seleno-methionine			Garlic as and primary metabolic product of SeMet	Antioxidant, regulating sele-noprotein TRXR-1 [243]
74-81-7	Caprylate			Metabolites	In lacking nitrogen on *C. elegans*: 7.0% increase in mean lifespan and needs further research [51]
6893-26-1	d-Glutamate			Amino acids	18.0–114.0% increase in mean lifespan and needs more research [103]
10257-28-0	Galact-opyranose			Sugars metabolites	In lacking nitrogen on *C. elegans*: 6.0% increase in mean lifespan and needs more research [103]
56-40-6	Glycine			Amino acids	10.0% increase in mean lifespan and needs more research [103]
6027-13-0	Homocysteine			Nitrogen containing metabolites	13.0% increase in mean lifespan and needs more research [103]
87-89-8	Inositol			Metabolites	In lacking nitrogen on *C. elegans:* 17.0% increase in mean lifespan and needs more research [103]
320-77-4	Isocitrate			TCA cycle intermediate	13.0% increase in mean lifespan and needs more research [103]
7004-09-3	Isoleucine			Amino acids	3.0% increase in mean lifespan and needs more research [103]
70-26-8	Ornithine			Amino acids	8.0% increase in mean lifespan and needs more research [103]
138-08-9	Phosphoenol-pyruvate			Metabolites	In lacking nitrogen on *C. elegans*: 12.0% increase in mean lifespan and needs more research [103]
98-98-6	Picolinic acid			Endogenous metabolite of the kynurenine pathway	7.0% increase in mean lifespan and needs further research [103]
10257-32-6	Ribopyranose			Sugars metabolites	In lacking nitrogen on *C. elegans*: 9.0% increase in mean lifespan and needs more research [103]
56-14-4	Succinate			TCA cycle intermediates	11.0% increase in mean lifespan and needs more research [103]
72-19-5	Threonine			Amino acids	8.0% increase in mean lifespan and needs more research [103]
72-18-4	Valine			Amino acids	13.0% increase in mean lifespan and needs more research [103]
58-86-6	Xylose			Sugars metabolites	In lacking nitrogen on *C. elegans*: 6.0% increase in mean lifespan and needs more research [103]
With anti-aging activities in other aging models					
32619-42-4	Oleuropein			*Olea europea* leaf	In cell: 15.0% increase in mean lifespan; and increasing proteasome-mediated degradation rates, retaining proteasome function and Nrf2/heme oxygenase-1 pathway [244]
84605-18-5	Cyclo-astragenol			*Astragalus membranaceus*	In PC12 cells and primary neurons: inducing telomerase activity and cAMP response element binding (CREB) [245]
528-58-5	Cyanidin			Fruits and vegetables	In cell: antioxidant, decreasing expressions of nuclear factor-kappaB, cyclooxygenase-2, and nitric oxide synthase [246]
88095-77-6	Dieckol			*Eckloina cava*	In radiation-induced cell damages: protecting effects on UV-B [247]
1229519-12-3	HDTIC-1			Herb *Astragalus membranaceus* var. *mongholicus*	In cell: antioxidant, improving proliferation, inhibiting glycation end product formation, slowing down telomere shortening rate [248, 249]
1229519-13-4	HDTIC-2			Herb *Astragalus membranaceus* var. *mongholicus*	In cell: antioxidant, improving proliferation, inhibiting glycation end product formation, slowing down telomere shortening rate [248, 249]
87798-94-5	Quercetin caprylate			Quercetin derivative	In cell: antioxidant, proteasome activator [250]
501334-35-6	Collemin A			Lichenized ascomycete *Collema cristatum*	In cell and human skin: preventing pyrimidine dimer formation and UV-B induced erythema [251]
87425-34-1	Nolinospiroside F			*Ophiopogon japonicus*	In *S. cerevisiae*: 23.0% increase in mean lifespan and antioxidant [252, 253]
57103-57-8	(−)-Glyceollin I			Soybeans	In *S. cerevisiae*: calorie restriction mimetic [254]
487-52-5	Butein			*Toxicodendron vernicifluum*	In *S cerevisia*: 31.0% increase in mean lifespan and regulating Sir2 [255]
1341-23-7	Nicotinamide riboside			NAD(+) precursor	In *S. cerevisiae*: 20.0% increase in mean lifespan and increasing net NAD(+) synthesis and Sir2 function [252, 253]
434-13-9	Lithocholic acid			Major bile acids excreted by mammals	In *S. cerevisiae*: 100.0% increase in mean lifespan and modulating housekeeping longevity assurance processes [256, 257]
57-94-3	Curare			*Chondrodendron tomentosum, Menispermaceae* or *Strychnos*	In *Asplanchna brightwelli*: 34.0% increase in mean lifespan and needs further research [258]

**Table 2:** Natural product extracts with anti-aging activities

Complex or extracts	Source	Anti-aging activity and proposed anti-aging mechanism
With anti-aging activities in two aging models		
Green tea extract	Green tea	In mice: 7.0% increase in mean lifespan and antioxidant [44] In *D. melanogaster*: 16.0% increase in mean lifespan and antioxidant [96]
Korean mistletoe water extract	*Viscum album coloratum*	In *D. melanogaster:* 20.0% increase in mean lifespan and regulating Sir2 [259] In *C. elegans*: 10.0% increase in mean lifespan and antioxidant [259]
With anti-aging activities in rats or mice		
A-type proanthocyanidins-rich cranberry extract	Cranberry	In mice: antioxidant [260]
*Fungus Phellinus* sp. polysaccharide	*Fungus Phellinus* sp.	In mice: antioxidant [261]
Polysaccharides of *Dicliptera chinensis* (L.) Juss	*Dicliptera chinensis* (L.) Juss	In mice: scavenging free radical and antioxidant [262]
Polysaccharides of *Urtica*	*Urtica*	In d-galactose-induced mice: antioxidant [263]
Cocoa polyphenolic extract	Acticoa powder	In rats: 11.0% increase in mean lifespan and retarding age-related brain impairments [264]
*Nigella Sativa* fixed oil	*Nigella Sativa*	In mice: reducing lipid peroxidation, Bax/Bcl2, and *caspase*-*3* [265]
Exopolysaccharides of *Agrocybe*	*Agrocybe cylindracea*	In d-galactose-induced mice: antioxidant, reducing the contents of malonaldehyde (MDA) and total cholesterol (TC) [266]
Neem leaves extract	Neem	In UVB-irradiated NHDFs, hairless mice: increasing TGF-β1, decreasing AP-1, ROS, and MAPK [267]
With anti-aging activities in *Drosophila melanogaster*		
APPLE polyphenols	Apple	10.0% increase in mean lifespan and antioxidant [268]
Cocoa		~14.0% increase in mean lifespan and antioxidant [269]
*Cordyceps sinensis* oral liquid	Traditional Chinese medicine	32.0% increase in mean lifespan and antioxidant [270]
*Cynomorium songaricum* Rupr	Traditional Chinese medicine	15.0% increase in mean lifespan and antioxidant [271]
*Emblica officinalis* (fruit)	*Emblica officinalis*	6.0% increase in mean lifespan and antioxidant [272]
Rhizome powder of Rhodiola rosea	Rhodiola rosea	17.0% increase in mean lifespan and antioxidant [273]
*Curcuma longa* (*rhizome*)	*Curcuma longa*	18.0% increase in mean lifespan and antioxidant [272]
Oregano and cranberry extracts	Oregano and cranberry	~43.0% in male and ~62.0% in female (full diet +2% OC) increase in mean lifespan and partly through DR-independent pathways [274]
Cinnamon extract	Cinnamon	17.0% in male, 37.0% in female increase in mean lifespan and regulating insulin signaling [275]
*Ludwigia octovalvis* extract	*Ludwigia octovalvis*	24.0% increase in mean lifespan and regulating AMPK [276]
Jujube fruit	Jujube	11.1% increase in mean lifespan and regulating FoxO [277]
Black tea extract	Black tea	21.4% increase in mean lifespan and inhibiting the ageing-related accumulation of iron [278]
Cranberry anthocyanin extract	Cranberry	10.0% increase in mean lifespan and up-regulation of SOD1 and down-regulation of MTH, InR, TOR and PEPCK [279]
*Rosa damascena* extract	*Rosa damascena*	32.0% increase in mean lifespan and increasing sensitivity to heat [280]
With anti-aging activities in *Caenorhabditis elegans*		
*Acanthopanax sessiliflorusstem* stem extract	*Acanthopanax sessiliflorusstem* stem	16.8% increase in mean lifespan and antioxidant [281]
*Angelica sinensis* peptides	*Angelica sinensis*	~20.0% increase in mean lifespan and antioxidant [282]
Apple procyanidins	Apple	12.1% increase in mean lifespan and antioxidant [283]
Blueberry polyphenols	Blueberry	28.0% increase in mean lifespan and antioxidant [284]
Extract from seed of *Platycladus orientalis*	*Platycladus orientalis*	24.5% increase in mean lifespan and antioxidant [285]
Ginko biloba extract	Ginko biloba	8.0–25.0% increase in mean lifespan and antioxidant [286]
HonTsai Tai extract	HonTsai Tai	8.0% increase in mean lifespan and antioxidant [287]
KPG-7	*Herb mixture*	12.0% increase in mean lifespan and antioxidant [288]
*Panax notoginseng* Polysaccharides	*Panax notoginseng*	21.0% increase in mean lifespan and antioxidant [289]
*Tenebrio molitor* extracts	*Tenebrio molitor*	30.6% increase in mean lifespan and antioxidant [290]
Erchen wan	Traditional Chinese medicine	22.0% increase in mean lifespan and antioxidant [291]
Huanshao dan	Traditional Chinese medicine	38.0% increase in mean lifespan and antioxidant [291]
Liuwei dihuang wan	Traditional Chinese medicine	13.0% increase in mean lifespan and antioxidant [291]
Shengmai yin	Traditional Chinese medicine	47.0% increase in mean lifespan and antioxidant [291]
Shiquan dabu wan	Traditional Chinese medicine	15.0% increase in mean lifespan and antioxidant [291]
*Bletilla striata* polysaccharide	*Bletilla striata*	~20.0% increase in mean lifespan and regulating IIS pathway [292]
Ethylacetate fraction from *Ribes fasciculatum*	*Ribes fasciculatum*	16.3% increase in mean lifespan and regulating IIS pathway and SIR-2.1 [293]
Peptides from sesame cake	Sesame cake	15.6% increase in mean lifespan and regulating SKN-1signaling [294]
*Astragalus membranaceus* polysaccharide	*Astragalus membranaceus*	24.0% increase in mean lifespan and regulating DAF-16 [295]
Garlic extract	Garlic extract	21.0% increase in mean lifespan and regulating DAF-16 [296]
Reishi mushroom polysaccharide	Reishi mushroom	~20.0% increase in mean lifespan and regulating TIR-1 and DAF-16 [99]
Royal Jelly	Honeybee	18.0% increase in mean lifespan and DAF-16 dependent [297, 298]
Ayurvedic polyherbal extract	Ayurvedic	16.1% increase in mean lifespan and regulating DAF-2, SKN-1, SOD-3, GST-4, and DAF-16 [299]
*Damnacanthus officinarum* leaf extract	*Damnacanthus officinarum*	19.0% increase in mean lifespan and regulating neuroprotective activity [300]
Deuterohemin peptide	Peptides	21.0% increase in mean lifespan and antioxidant, regulating DR [301]
*Eleutherococcus senticosus* root extract	*Eleutherococcus senticosus*	16.0% increase in mean lifespan and antioxidant, regulating DAF-16 [302]
Lowbush cranberry	Lowbush cranberry	22.0% increase in mean lifespan and altering mechanosensory neuron aging [303]
Mulberry leaf polyphenols	Mulberry leaf	23.0% increase in mean lifespan and regulating DAF-12, PHA-4, NHR-80, and DAF-16 [304]
Dauer-inducing Pheromone	Worms	27.0% increase in mean lifespan and needs more research [305]
With anti-aging activities in other aging models		
Annurca apple extracts	Annurca apple	In *S. cerevisiae*: antioxidant, antiapoptotic [306]
Red algal extracts	Red algal	In *Brachionus manjavacas*: 9.0% increase in mean lifespan and needs further research [307]

**Table 3:** Clinical medicine with anti-aging activities

CAS	Chemicals	Structure	Clinical application	Anti-aging activity and proposed anti-aging mechanism
With anti-aging activities in a variety of aging models				
58-08-2	Caffeine		Psychoactive drug	In rats: antioxidant, alleviating neuroinflammation and neurodegeneration [75] In *zebrafish:* 29.4% increase in mean lifespan and regulating proteostasis [74] In *C. elegans*: 29.4% increase in mean lifespan and regulating IIS pathway and proteostasis [73]
657-24-9	Metformin		Treatment of type 2 diabetes and polycystic ovary syndrome	In rats: altering erythrocyte redox status [70] In *D. melanogaster:* has not effect on fecundity or lifespan and activating AMPK, reducing lipid stores [72] In *C. elegans*: 40.0% increase in mean lifespan and regulating AMPK, LKB1, and SKN-1 [71]
53123-88-9	Rapamycin		Used to coat coronary stents, prevent organ transplant rejection and to treat a rare lung disease called lymphangioleiomyomatosis	In mice: 14.0% increase in mean lifespan for females and 9% for males and reducing mTOR activity [76–82] In *D. melanogaster:* 13.0% increase in mean lifespan and regulating TORC1 branch of the TOR pathway, through alterations to both autophagy and translation [83] In *C. elegans*: 19.0% increase in mean lifespan and regulating TOR, SKN-1 and DAF-16 [84]
With anti-aging activities in two aging models				
50-78-2	Aspirin		Used to treat pain, fever, and inflammation	In genetically heterogeneous male mice: 8.0% increase in mean lifespan and needs further research [135] In *C. elegans*: 30.0% increase in mean lifespan and antioxidant, regulating AMPK and insulin-like signaling pathway [183, 308]
2086-83-1	Berberine		Used to treat bacillary dysentery and gastroenteritis	In aged mice: suppressing neuroinflammation, reducing vascular stiffness in aged mice through suppression of TRPV4 [309, 310] In *D. melanogaster:* 46.0% increase in mean lifespan and inhibiting kynurenine (KYN) formation from tryptophan (TRP) [311]
102518-79-6	Huperzine A		Treatment for neurological conditions such as Alzheimer’s disease	In d-galactose-induced mice: inhibiting DAMPs-mediated NF-κB nuclear localization and activation [312] In *C. elegans*: 14.0% increase in mean lifespan and needs further research [285]
10118-90-8	Minocycline		Antibiotic	In *D. melanogaster*: 63.0% increase in mean lifespan and antioxidant [313] In *C. elegans*: 29.0% increase in mean lifespan and antioxidant [176]
114-86-3	Phenformin		Antidiabetic	In mice: 21.0% increase in mean lifespan and decreasing the body weight, slowing down the age-related decline of the reproductive function in female rats [314] In *C. elegans*: 29.0% increase in mean lifespan and needs further research [315]
59-02-9	Vitamin E		Vitamins	In rats: reducing the oxidative stress increased in old age [316, 317] In *C. elegans*: 23.0% increase in mean lifespan and antioxidant [318]
With anti-aging activities in rats or mice				
692-13-7	Buformin		Antidiabetic	In rats: 7.0% increase in mean lifespan in female and decreasing the body weight, slowing down the age-related decline of the reproductive function in female rats [314]
73-31-4	Melatonin		Regulating sleep and wakefulness	In male Wistar: restoring rSocs1 rhythms and levels in various tissues [319]
155974-00-8	Ivabradine		Used for the symptomatic management of stable heart related chest pain and heart failure	In C57BL/6 J mice: 6.0% increase in mean lifespan and reducing heart rate [320]
56180-94-0	Acarbose		Antidiabetic	In SAMP8 mice and male mice: 22.0% increase in mean lifespan and changing in the insulin system and the levels of BDNF, IGF-1R, and the pre-synaptic proteins Syt1 and Stx1 [321, 322]
51384-51-1	Metoprolol		Used to treat high blood pressure, chest pain due to poor blood flow to the heart, and a number of conditions involving an abnormally fast heart rate	In mice: 10.0% increase in mean lifespan and needs further research [323]
99200-09-6	Nebivolol		Treatment of hypertension	In mice: 6.4% increase in mean lifespan and needs further research [323]
With anti-aging activities in *Drosophila melanogaster*				
13123-37-0	Riboflavin		Vitamin	14.1% increase in mean lifespan and increasing SOD1 and CAT, inhibiting LF [324]
84057-84-1	Lamotrigine		Anticonvulsant	17.0% increase in mean lifespan and reducing locomotor activity and metabolic rate [325]
1716-12-7	4-Phenyl-butyrate		Used to treat urea cycle disorder	40.0% increase in mean lifespan and increasing histone acetylation [326]
52757-95-6	Sevelamer		Used to treat hyperphosphatemia in patients with chronic kidney disease	16.0% increase in mean lifespan and regulating cellular and organismic phosphate levels [327]
79902-63-9	Simvastatin		Hypolipidemic	25.0% increase in mean lifespan and decreasing specific protein prenylation [328]
871700-17-3	Trametinib		Anti-cancer	12.0% increase in mean lifespan and inhibiting Ras-Erk-ETS signaling [329]
58880-19-6	Trichostatin A		Antifungal antibiotic	27.0% increase in mean lifespan and changing the level of histone acetylation, influencing the expression of *hsp22* gene [330]
57-27-2	Morphine		Treatment of acute pain and chronic pain	22.0% increase in mean lifespan and needs further research [331]
With anti-aging activities in *Caenorhabditis elegans*				
14028-44-5	Amoxapine		Antidepressant	33.0% increase in mean lifespan and antioxidant [176]
298-57-7	Cinnarizine		Treatment of vertigo, motion sickness, and vomiting	15.0% increase in mean lifespan and antioxidant [176]
59865-13-3	Cyclosporin A		Immunosuppressants	18.0% increase in mean lifespan and antioxidant [176]
427-51-0	Cyproterone acetate		Antiandrogen and progestogen	23.0% increase in mean lifespan and antioxidant [176]
17230-88-5	Danazol		Treatment of endometriosis	13.0% increase in mean lifespan and antioxidant [176]
127-33-3	Demeclocycline hydrochloride		Antibiotic	16.0% increase in mean lifespan and antioxidant [176]
564-25-0	Doxycycline		Antibiotic	18.0% increase in mean lifespan and antioxidant [176]
10592-13-9	Doxycycline hydrochloride		Antibiotic	18.0% increase in mean lifespan and antioxidant [176]
119431-25-3	Eliprodil		NMDA antagonist, treatment of acute ischemic stroke	16.0% increase in mean lifespan and antioxidant [176]
23256-50-0	Guanabenz acetate		Antihypertensive	12.0% increase in mean lifespan and antioxidant [176]
29110-48-3	Guanfacine hydrochloride		Treatment of hyperactivity	15.0% increase in mean lifespan and antioxidant [176]
27833-64-3	Loxapine succinate		Antipsychotic	43.0% increase in mean lifespan and antioxidant [176]
57149-08-3	Naftopidil di-hydrochloride		Antihypertensive	14.0% increase in mean lifespan and antioxidant [176]
54527-84-3	Nicardipine hydrochloride		Used to treat high blood pressure and angina	23.0% increase in mean lifespan and antioxidant [176]
39562-70-4	Nitrendipine		Used in the treatment of primary (essential) hypertension to decrease blood pressure and can reduce the cardiotoxicity of cocaine	25.0% increase in mean lifespan and antioxidant [176]
894-71-3	Nortriptyline hydrochloride		Tricyclic antidepressant	21.0% increase in mean lifespan and antioxidant [176]
60607-34-3	Oxatomide		Anti-allergic	25.0% increase in mean lifespan and antioxidant [176]
130-61-0	Thioridazine hydrochloride		Antipsychotic	31.0% increase in mean lifespan and antioxidant [176]
2068-78-2	Vincristine sulfate		Anti-cancer	12.0% increase in mean lifespan and antioxidant [176]
97-59-6	Allantoin		Used to treat gastric ulcer, duodenal bulb ulcer, chronic gastritis	21.9% increase in mean lifespan and caloric restriction mimetics [332]
169590-42-5	Celecoxib		COX-2 selective nonsteroidal anti-inflammatory drug (NSAID) It is used to treat the pain and inflammation of osteoarthritis, rheumatoid arthritis, ankylosing spondylitis, acute pain	19.0% increase in mean lifespan and inhibiting insulin-like signaling [333]
99-66-1	Valproic acid		Used to treat epilepsy and bipolar disorder and to prevent migraine headaches	35.0% increase in mean lifespan and regulating IIS pathway [334]
103-90-2	Acetaminophen		Used to treat pain and fever	49.0% increase in mean lifespan and regulating CBP-1 [224]
69-52-3	Ampicillin		Antibiotic	34.0% increase in mean lifespan and antimicrobial [284]
41859-67-0	Bezafibrate		Treatment of hypertriglyceridemia	13.0% increase in mean lifespan and regulating NHR-49/PPARalpha-dependent manner [335]
637-07-0	Clofibrate		Lipid-lowering agent used for controlling the high cholesterol and triacylglyceride level in the blood	16.0% increase in mean lifespan and regulating NHR-49/PPARalpha-dependent manner [335]
49562-28-9	Fenofibrate		Used to reduce cholesterol levels in people at risk of cardiovascular disease	19.0% increase in mean lifespan and regulating NHR-49/PPARalpha-dependent manner [335]
127-48-0	Trimethadione		Anticonvulsant	47.0% increase in mean lifespan and regulating neuromuscular activity [336]
42971-09-5	Vinpocetine		Treatment of cerebrovascular disorders and age-related memory impairment	15.0% increase in mean lifespan and regulating PDE1 [176]
59-30-3	Folic acid		Used to treat anemia caused by folic acid deficiency	27.0% increase in mean lifespan and antioxidant, regulating SIR-2.1, SKN-1, and DAF-16 [337]
77-67-8	Ethosuximide		Used to treat absence seizures	17.0% increase in mean lifespan and disrupting sensory function, regulating DAF-16 [336, 338, 339]
50264-69-2	Lonidamine		Anti-cancer	8.0% increase in mean lifespan and promoting longevity in a *pmk*-*1*sensitive manner by increasing formation of ROS [340]
50-55-5	Reserpine		Antipsychotic, and antihypertensive	31.0% increase in mean lifespan and antioxidant, modulating acetylcholine release [341, 342]
13292-46-1	Rifampicin		Antibiotic	56.0% increase in mean lifespan and reducing advanced glycation end products and activating DAF-16 [343]
6998-60-3	Rifamycin SV		Antibiotic	21.0% increase in mean lifespan and reducing advanced glycation end products and activating DAF-16 [343]
723-46-6	Sulfa-methoxazole		Antibiotic	34.0% increase in mean lifespan and increasing lipid peroxidation oxidative stress [344]
56-75-7	Chloram-phenicol		Antibiotic	16.0% increase in mean lifespan and needs further research [345]
With anti-aging activities in other aging models				
53-06-5	Cortisone		Used to reduce inflammation and attendant pain and swelling at the site of the injury	In *Asplanchna brightwelli*: 21.0% increase in mean lifespan and stabilizing lysosomal membranes, or altering resource allocation by the rotifers [346]
35891-70-4	Myriocin		Antibiotic ISP-1 and thermozymocidin	In *S. cerevisiae*: activating the Snf1/AMPK pathway, down-regulating the protein kinase A (PKA) and target of rapamycin complex 1 (TORC1) pathways [347]

**Table 4:** Synthetic compounds with anti-aging activities

CAS	Chemicals	Structure	Anti-aging activity and proposed anti-aging mechanism
With anti-aging activities in two aging models			
51-28-5	2,4-Dinitrophenol		In mice: 7.0% increase in mean lifespan; enhancing tissue respiratory rates, improving serological glucose, triglyceride and insulin levels, decreasing reactive oxygen species levels and tissue DNA and protein oxidation, as well as reduced body weight [348] In *D. melanogaster*: 20.0% increase in mean lifespan; increasing the rate of oxygen consumption by isolated mitochondria and tissue homogenates, decreasing the activity of alcohol dehydrogenase [349]
With anti-aging activities in mice			
91-53-2	Ethoxyquin		In C3H mice: 18.0% increase in mean lifespan in male, 20.0% in female and antioxidant [350]
1001645-58-4	SRT1720		In mice: 9.0% increase in mean lifespan and inhibiting proinflammatory gene expression [351]
With anti-aging activities in *Drosophila melanogaster*			
307297-39-8	Epitalon		17.0% increase in mean lifespan and antioxidatant [352]
34592-47-7	Thiazolidine carboxylic acid		31.0% increase in mean lifespan and antioxidatant [353]
133550-30-8	AG-490		18.0% increase in mean lifespan and activating ERK1/2 signaling [354]
4431-00-9	Aurintri-carboxylic acid		15.0% increase in mean lifespan and regulating p66ShcA [355]
91742-10-8	HA-1004 (dihydrochloride)		18.0% increase in mean lifespan and inhibiting protein kinase [354]
103745-39-7	HA-1077 (Fasudil)		15.0% increase in mean lifespan and inhibiting protein kinase [354]
5108-96-3	Pyrrolidine dithiocarbamate		16.0% increase in mean lifespan and inhibiting NF-κB [356]
With anti-aging activities in *Caenorhabditis elegans*			
75529-73-6	Amperozide hydrochloride		38.0% increase in mean lifespan and antioxidatant [176]
193611-72-2	BRL 15572		10.0% increase in mean lifespan and antioxidatant [176]
433695-36-4	BRL 50481		18.0% increase in mean lifespan and antioxidatant [176]
145915-58-8	DAPH (4,5-dianilino-phthalimide)		15.0% increase in mean lifespan and antioxidatant [176]
53177-12-1	EUK-8		54.0% increase in mean lifespan and antioxidant [357]
81065-76-1	EUK-134		54.0% increase in mean lifespan and antioxidant [357]
98299-40-2	Hexahydro-sila-diphenidol		15.0% increase in mean lifespan and antioxidatant [176]
142273-20-9	Kenpaullone		27.0% increase in mean lifespan and antioxidatant [176]
83846-83-7	Ketanserin tartrate		13.0% increase in mean lifespan and antioxidatant [176]
13614-98-7	Minocycline hydrochloride		43.0% increase in mean lifespan and antioxidatant [176]
66104-23-2	Pergolide methanesulfonate		37.0% increase in mean lifespan and antioxidatant [176]
497-27-8	4-Phenyl-3-Furoxan-carbonitrile		30.0% increase in mean lifespan and antioxidatant [176]
58-33-3	Promethazine hydrochloride		32.0% increase in mean lifespan and antioxidatant [176]
7681-67-6	Propionyl-promazine hydrochloride		20.0% increase in mean lifespan and antioxidatant [176]
	Trans-3,5-dimethoxy-4-fluoro-4-hydroxystilbene		3.6% increase in mean lifespan and antioxidatant [358]
	Trans-2,4,5-trihydroxystilbene		5.4% increase in mean lifespan and antioxidatant [358]
78416-81-6	Trequinsin hydrochloride		27.0% increase in mean lifespan and antioxidatant [176]
274-85-1	1,2,4-Triazolo[1,5-a]pyridine		12.0% increase in mean lifespan and antioxidant [359]
138090-06-9	(*R*,*R*)-*cis*-Diethyl- tetrahydro-2,8-chrysenediol		7.0% increase in mean lifespan and increasing stress resistance [176]
2390-54-7	Thioflavin T		60.0% increase in mean lifespan and regulating HSF-1 and SKN-1 [360]
175698-05-2	3,3-Diethyl-2-pyrrolidinone		31.0% increase in mean lifespan and regulating neuromuscular activity [336]
631-64-1	Dibromoacetic acid		15.0% increase in mean lifespan and inducing protective stress response [225]
82-76-8	*N*-Phenyl periacid(ANSA)		22.7% increase in mean lifespan and increasing aging related pharyngeal pumping rate [63]
51314-51-3	Benzimidazole derivative M084		19.10% increase in mean lifespan; regulating IIS pathway, AMPK, SIR-2.1, SKN-1, mitochondrial electron transport chain, and mitochondrial unfolded protein response [361–364]
With anti-aging activities in *Asplanchna brightwelli*			
111-17-1	3,3′-Thiodipropionic acid		16.0% increase in mean lifespan and increasing lipid peroxides [365]

Among the 55 complex or extracts from natural products, 8, 14 and 29 of them were tested in mice, fruit fly and *C. elegans*, respectively. A majority of these extracts present antioxidative activity.

Among the 62 clinical medicine with anti-aging activity, three (rapamycin, metformin, caffeine) present anti-aging activities in three aging models, six (aspirin, berberine, huperzine A, minocycline, phenformin, and vitamin E) in two aging models, two (buformin and melatonin) in rats, four (ivabradine, acarbose, metoprolol, and nebivolol) in mice, 8 in *D. melanogaster*, 37 in *C. elegans*, cortisone in *Asplanchna brightwelli* and myriocin in *S. cerevisiae*, respectively. Interestingly, the anti-aging mechanisms of the most drugs are different from their clinical applications.

We also summarized 35 synthetic compounds with explicit anti-aging activity (Table 4). 2,4-Dinitrophenol presents anti-aging activities in mice and fruit fly, ethoxyquin and SRT1720 in mice. Seven and 24 compounds present anti-aging activity in fruit fly and *C. elegans*, respectively. 3,3′-thiodipropionic acid with anti-aging activity in *Asplanchna brightwelli*. Twenty-one of the 35 compounds present antioxidative activity.

In total, there are 212 and 46 compounds present anti-aging activity in *C. elegans* and fruit fly, respectively, indicating *C. elegans* and fruit fly are the most popular aging models for anti-aging screening. Those compounds present anti-aging activity in both *C. elegans* and fruit fly are worth to be further investigated in mammalian models.

## Prospects of Discovering Anti-aging Molecules from Natural Products

Many clinical medicines are derived from natural products. But in the past two decades, pharmaceutical companies have been enthusing the drug development strategy of high-throughput screening (HTS) and combinatorial synthesis of enormous synthetic libraries of small molecules. Natural products were largely neglected for unsuitable for HTS of targeted protein assay and difficult in compound isolation and synthesis. But the achievement of new lead discovery and new drug approval was disappointing [85]. Compared with synthetic compounds, natural products are secondary metabolite, evolutionarily optimized with biologically relevant chemical space and preferred ligand binding motif, are not only biologically active, but with a high degree of bioavailability, suitable for functional and phenotypic assays [86]. Recent innovation in techniques for structural elucidation, metabolomics for profiling and isolation, and metagenomics or gene manipulation for synthetic pathways has facilitated to explore the enormous biodiversity on earth, including plant, microorganism and marine organism [87]. Engineered production of natural products from uncultivated species could extremely expand the chemical space of natural products by synthetic biology [88]. Moreover, modern computer-assisted drug design could utilize natural-product-derived fragments to computationally infer the biomolecular targets and activities of natural products and fragment-based de novo design. As summarized in above, currently discovered agents with anti-aging activity, majority of them are natural products. Therefore, natural products are invaluable sources and provide great promise for developing anti-aging medicine.
